# Impact of Apple Pomace Powder on the Bioactivity, and the Sensory and Textural Characteristics of Yogurt

**DOI:** 10.3390/foods11223565

**Published:** 2022-11-09

**Authors:** Liliana Popescu, Tatiana Ceșco, Angela Gurev, Aliona Ghendov-Mosanu, Rodica Sturza, Ruslan Tarna

**Affiliations:** Faculty of Food Technology, Technical University of Moldova, 9/9 Studentilor St., MD-2045 Chisinau, Moldova

**Keywords:** apple pomace powder, dietary fiber, yogurt, textural parameters, antioxidant activity, and quality

## Abstract

This study focused on the development of a yogurt with an improved structure, texture and antioxidant activity level, by using apple pomace (AP) powder that was obtained in large quantities during the production of juices. The objective was to determine the sensory, physicochemical, textural and antioxidant characteristics of yogurt with the addition of AP powder (0.2–1.0%), during its shelf life. The physicochemical composition of AP was determined as follows: dietary fibers—62.73%, including pectin—23.12%; and the content of the antioxidant compounds in AP—total polyphenols (728.8 mg GAE/100 g DW), flavonoids (246.5 mg QE/100 g DW), tannins (63.54 mg TAE/100 g DW), carotenoids (4.93 mg/100 g DW) and the ability to inhibit the free radical (2433 µmol TE/100 g DW). AP addition reduces the yogurt fermentation time. The increase in the total dietary fiber content of up to 0.63% and in the insoluble fiber of up to 0.14% was attested in this study, as well as a significant increase in antioxidant activity, which correlated to the AP content. The addition of AP improved the textural properties of the yogurt during storage (20 days) and led to a significant reduction in syneresis. The influence of the AP content and the storage period on the textural characteristics and the overall acceptability of the yogurt samples were analyzed by the mutual information method. The AP content greatly influenced the yogurt’s quality, with the information analysis value for the overall acceptability being 0.965 bits. The analysis of the sensory and textural parameters of the yogurt during storage (1–20 days) demonstrated that samples with AP in proportions of 0.6–0.8% were evaluated with the highest score.

## 1. Introduction

Food industries produce millions of tons of waste during processing, which becomes a significant environmental, economic and nutritional problem. Agricultural waste could serve as an important source of bioactive compounds, including antioxidants, dietary fibers, polysaccharides, vitamins, carotenoids, pigments and oils [1]. These compounds are of increasing scientific interest due to the benefits they bring to human life [2].

Among fruit wastes, apple pomace (AP) is a potential source of phytochemicals and contains significant amounts of carbohydrates, as well as small amounts of proteins, vitamins and minerals [3]. It has been estimated that the production of apple juice results in a product that is low in polyphenolic compounds and that has only 3–10% of the antioxidant activity of the fruit from which it is produced. Most of the polyphenol compounds remain in apple pomace—a heterogeneous mixture of peel, core, seed, stem and soft tissue [4]. Currently, there are few uses of AP, with most of it being used on farms as animal feed or transported to landfills and incinerators. Sad to say, this has a negative effect on the environment, contributing to the greenhouse effect [5].

AP contains insoluble sugars, including cellulose (127.9 g/kg dried weight (DW)); hemicellulose (7.2–43.6 g/kg DW); lignin (15.3–23.5 g/kg DW); and simple sugars, such as glucose, fructose and galactose [5,6]. In addition, AP is an important source of substances with high antioxidant activity, in particular, quercetin glycosides, phloridzin, phloretin, epicatechin, chlorogenic acid and other polyphenolic constituents [7,8,9,10]. Therefore, AP can be used in various food systems after minimal processing or in the form of extracts, significantly increasing the functional value of food and contributing to a reduction in food waste [6].

Yogurt plays an essential role in most dietary recommendations, providing nutrients and bioactive compounds that are essential for health, such as the following: vitamins and minerals in an easily assimilable form; lactose; bioactive proteins; lipids; and live lactic bacteria, which is beneficial to the gastrointestinal tract [11]. To meet consumer needs, new assortments of yogurt-based products are being investigated, leading to a steady increase in the sales and popularity of this dairy product [12].

Yogurt is a product that is characterized by an unstable structure, which is vulnerable to deformation by shearing, with only a slight recovery at rest or under the action of a low shearing speed [13]. The microstructure of milk protein gels and their rheological properties affect the texture, sensory properties and storage stability of yogurt [14]. The main factors that affect the structure and texture of yogurt are as follows: the protein and fat content, the technological process, and the amount and properties of the added ingredients [14,15,16,17].

The structure and texture of yogurt is controlled, most often, by the addition of stabilizers. The most common food stabilizers and thickeners are various polysaccharides, such as modified starch, xanthan, carrageenan, methyl and hydroxypropylmethyl cellulose, etc. [18]. These polysaccharides interact with the casein network and contribute to the formation of gels in biphasic systems (liquid–solid): a three-dimensional, continuous structure that forms the gel matrix, which holds a finely dispersed liquid phase [19]. Although these food additives are considered safe by regulatory agencies, they are perceived as harmful by consumers due to their unfamiliarity and the risk perception of chemicals [20].

Therefore, the objective of this study was to determine the sensory, physicochemical, textural and antioxidant characteristics of yogurt with the addition of AP powder, during its shelf life. This study focused on the development of a yogurt with an improved structure and texture, and improved antioxidant activity by using AP powder.

## 2. Materials and Methods

### 2.1. Materials

Golden Delicious apples were harvested during September 2021 from AgroProduct, Colicauti commune, Briceni, Republic of Moldova (48°18′36″ N 27°8′54″ E), which has orchard plantations of apples over 200 ha. Skimmed milk powder containing 35% protein, 50% lactose and 1% fat, according to the information on the label, was purchased from Inlac, Republic of Moldova. Freeze-dried, direct vat set (FD-DVS) yogurt starter culture contains *Streptococcus thermophilus*, *Lactobacillus delsbrueckii* subsp. *bulgaricus*, *Lactobacillus acidophilus* and *Bifidobacterium* (YAB 352B, Sacco, Italy). The 6-hydroxy-2,5,7,8-tetramethylchromane-2-carboxylic acid (Trolox) and the 1,1-diphenyl-2-picrylhydrazyl-hydrate (DPPH) were provided by Alpha Aesar (Haverhill, MA, USA). Aluminum chloride hexahydrate and the standard compounds (gallic acid (GA) and quercetin (QE)), were provided by Sigma-Aldrich (St. Louis, MO, USA). Folin–Ciocalteu phenol reagent was provided by Chem-Lab NV (Zedelgem, Belgium). Ethanol, n-hexane, methanol, sodium carbonate, diethyl ether, acetonitrile, chloroform, citric acid, potassium iodide, sodium thiosulfate, sodium hydroxide and potassium hydroxide—provided by Chemapol (Prague, Czech Republic)—were used. All reagents used in this study were of analytical grade. All spectrophotometric determinations were performed on a spectrophotometer UV-1900 (Shimadzu, Tokyo, Japan).

### 2.2. Production of Apple Pomace Powder

The AP was obtained after extracting the juice intended for the manufacture of apple cider and was blanched in citric acid solution with a concentration of 0.2%, at a temperature of 95 ± 2 °C, for 10 min, with the aim of inhibiting the oxidation processes responsible for the deterioration of the quality and colors of the pomace. It was then cooled to the temperature of 25 ± 1 °C, pressed, dried by forced convection in a laboratory oven, SLW 115 SMART (Pol-Eco Aparatura, Wodzisław Śląski, Poland), at a temperature of 60 ± 1 °C, to a moisture content of 7.8 ± 0.1% and crushed to a granularity of 70 ± 10 µm.

### 2.3. Apple Pomace Powder Characterization

#### 2.3.1. Physicochemical Analysis

Titratable acidity [21]; moisture and ash content [22]; fat content using Soxhlet extraction [23]; protein content [24]; total dietary fiber [25] and insoluble dietary fiber [26] were determined. Soluble solids content was determined with a digital refractometer, Kruss DR 201-95 (Kruss, Hamburg, Germany).

#### 2.3.2. Pectin Content

The pectin content in AP was determined by the method in [27], with some modifications. The extraction of pectin was carried out with an aqueous solution of citric acid at pH = 2.0 ± 0.1, the ratio AP:citric acid solution was 1:15 (*m/v*), at temperatures of 90 ± 2 °C, for 130 min, and then filtered. The supernatant was cooled to 4 ± 1 °C, 96% ethanol was added in a ratio of 1:1 (*v/v*) and filtered. The obtained precipitate was washed three times with 60% (*v/v*) hydroethanolic solution, vacuum dried at a temperature of 60 ± 1 °C to a moisture of 4.8 ± 0.1%, and weighed.

### 2.4. Apple Pomace Extract Characterization

#### 2.4.1. Production of Apple Pomace Extract

First, 0.5 g of AP powder was introduced into the volumetric flask and brought to the volume of 100 mL, with 50% (*v/v*) hydroethanolic solution. The prepared samples were further extracted by ultrasound-assisted method (ISOLAB Laborgeräte GmbH, Eschau, Germany) at a frequency of 37 kHz, temperature of 40 ± 1 °C, for 30 min. Afterwards, the samples were centrifuged at 4000 rpm for 10 min and the supernatant was collected and analyzed.

#### 2.4.2. Total Polyphenols and Flavonoids by Folin–Ciocalteu

The total polyphenol content (TPC) and the total flavonoid content (TFC) were determined according to [28], with some modifications. In the case of TPC, a 20 μL aliquot of extract sample was transferred to the test tube, and 6.5 mL of deionized water was added and vortexed for 10 s. Next, 500 μL of Folin–Ciocalteau reagent was added to the mixture and shaken for 10 s. The solution was incubated for 8 min at room temperature. Then, 3 mL Na_2_CO_3_ solution 10% (*w/v*) was added, stirred for 10 s and placed in a water bath, with stirring at temperature of 40 ± 1 °C, for 30 min. The results were calculated with a calibration curve using gallic acid (0–500 mg/L, R^2^ = 0.9980), and expressed in equivalents of gallic acid per 100 g of dried weight (DW) of AP powder (mg GAE/100 g DW).

For TFC determination, 1.5 mL of AlCl_3_ solution 2% in methanol (*w/v*) was added to 1.5 mL of extract and incubated at room temperature for 40 min. The TFC was calculated with a calibration curve using quercetin (0–160 mg/L, R^2^ = 0.9972). The results were expressed in milligrams equivalent of quercetin per 100 g of dried weight (DW) of AP powder (mg QE/100 g DW).

#### 2.4.3. Total Tannins by Folin–Ciocalteu

The total tannins were determined by Waterman and Mole (1994) [29], using the Folin–Ciocalteu reagent. The results were calculated with a calibration curve using tannic acid (0–50 mg/L, R^2^ = 0.9985) and expressed in milligrams equivalent of tannic acid per 100 g of dried weight (DW) of AP powder (mg TAE/100 g DW).

#### 2.4.4. Total Carotenoids

The total carotenoids content (TCC) was determined following the method described by Ghendov-Mosanu et al. (2020) [30] and Britton et al. (1995) [31]. A mixture of methanol/ethyl acetate/petroleum ether (1:1:1, *v/v/v*) was used for analysis. After filtering the extract, the residue was re-extracted twice, using the same solvent mixture. TCC was measured at the wavelength of maximum absorbance (λ_max_ = 450 nm). The experiments were conducted in low light. The TCC was calculated according to formula (1), as follows:

(1) $$\mathrm{TCC}\;(\mathrm{mg}/g)=\frac{A_{450}\cdot V\cdot D\cdot 1000}{2500\cdot 100\cdot m}$$

where, A_450_ is the mean absorbance maximum; V is the volume of the extract, mL; D is a dilution factor; 2500 is the absorption coefficient of carotenoids; and m is sample mass, g.

#### 2.4.5. Antioxidant Activity by Reaction with DPPH Radical

The method described by Brand-Williams et al. (1995) [32] was used to determine antioxidant activity (AA). The results were expressed in µmol trolox equivalent (TE) per 100 g of dried weight (DW) of AP powder (µmol TE/100 g DW), with a calibration curve (0–500 µmol/L, R^2^ = 0.9992) with trolox.

### 2.5. Production of Yogurt with Apple Pomace (YAP) Powder

The yogurt samples were obtained using 0.2 0.4, 0.6, 0.8 and 1% AP powder (0.2%YAP, 0.4%YAP, 0.6%YAP, 0.8%YAP and 1.0%YAP). Skimmed milk powder was mixed with distilled water (15 g/100 mL), to which different concentrations of AP powder were added (0.2%, 0.4%, 0.6%, 0.8% and 1.0%, relative to the reconstituted milk). The obtained mixtures were left at room temperature for 2 h for hydration, pasteurized at temperatures of 85 ± 1 °C for 10 min, cooled to temperatures of 39 ± 1 °C, inoculated with starter culture (0.02 U/1000 mL) and fermented at temperatures of 39 ± 1 °C to pH 4.60 ± 0.01. After fermentation, the yogurt was mixed, distributed in glass containers (125 g) and cooled to temperatures of 4 ± 1 °C.

### 2.6. Yogurt with Apple Pomace Powder Characterization

#### 2.6.1. Physicochemical Analysis

Fat content was determined by gravimetric methods [33]. Dry matter content was determined by ISO 6731:2010 [34]. Total dietary fibers and insoluble dietary fibers were determined according to the enzymatic–gravimetric method [25,26]. The pH was measured with a digital pH-meter (Mettler Toledo, Columbus, OH, USA), at 20 °C. Physicochemical properties, except of pH, were determined only on the first day of storage.

#### 2.6.2. Color Analysis

Instrumental color analysis of yogurt samples was performed using a Chroma Meter CR-400 (Konica Minolta, Japan). The CIELab color scale was used to obtain the luminance (L*), red/green component (a*) and yellow/blue component (b*) values. The total color differences (ΔE* ), between samples were calculated according to the formula in [35], as follows:

(2) $${\Delta E}^{*}=\sqrt{{\left(L_{i}^{*}-L_{o}^{*}\right)}^{2}+{\left(a_{i}^{*}-a_{o}^{*}\right)}^{2}+{\left(b_{i}^{*}-b_{o}^{*}\right)}^{2}},\;$$

where, $L_{o}^{*}$, $a_{o}^{*}$ and $b_{o}^{*}$ are the values of the control sample and $L_{i}^{*}$, $a_{i}^{*}$ and $b_{i}^{*}$ are the values of the yogurt sample with the addition of AP. ${\Delta E}^{*}$ comparisons were in relation to the color obtained at the control sample. The color of the yogurt samples was determined only on the first day of storage.

#### 2.6.3. Antioxidant Analysis by Reaction with DPPH Radical

First, 10 g of the yogurt sample was weighed into a 100 mL, quick-fit conical flask. Then, 20 mL of methanol:water, at 80:20 (*v/v*), were added. After shaking in ultrasonic water bath for 30 min, the residual solution was filtered in 25 mL measuring flask and completed to 25 mL by extraction solvent. The final extracts were collected separately in sealed glass containers and used for antioxidant analysis [36]. The AA of yogurt was determined using the method described by Brand-Williams et al. (1995) [32]. The results were expressed in µmol trolox equivalent (TE) per 100 g of yogurt (µmol TE/100 g). The AA of the yogurt samples was only determined on the first day of storage.

#### 2.6.4. Sensory Analysis

The sensory analysis of the yogurt samples was determined using the 5-points scoring scale, according to ISO 22935-3:2009 [37], by a panel of 9 assessors who were selected according to ISO 8586:2012 [38]. Appearance and consistency, color, odor and taste were evaluated. In scoring each property, the numerical discrete interval scale was used, as follows: 5 points—no deviation from the pre-established sensory specification; 4 points—minimal deviation from the pre-established sensory specification; 3 points—noticeable deviation from the pre-established sensory specification; 2 points—considerable deviation from the pre-established sensory specification and 1 point—very considerable deviation from the pre-established sensory specification. Sensory characteristics of the yogurt must correspond to the quality requirements for milk and dairy products [39] (Table 1).

Overall acceptability of the yogurt samples was expressed by the total score given by the panel of assessors. To calculate the total score, the average scores per sensory property were added and divided by the number of sensory properties. The sensory analysis of the yogurt samples was determined at different storage periods (1, 7, 14, 17 and 20 days).

#### 2.6.5. Texture Profile Analysis

Texture Profile Analysis (TPA) of the yogurt samples were analyzed with a TA.HD Plus C Texture Analyzer (Stable Micro Systems, Godalming, UK), according to Yilmaz-Ersan et al. (2019) [35]. TPA was determined at the storage temperature of the yogurt samples by the double-bite compression test, using an extrusion cylinder with a diameter of 40 mm, penetration distance 20 mm, pre-test speed 100 m/s, test speed 5 m/s, post-test speed 5 m/s, trigger force 5.0 g, data acquisition rate 2000 pps and 5 kg load cell. Firmness, springiness, cohesiveness, adhesiveness and gumminess were calculated from the obtained profiles using the software provided by Stable Micro Systems (Godalming, UK). Textural parameters were determined on the 1st, 7th, 14th, 17th and 20th days.

#### 2.6.6. Syneresis

Syneresis was determined by centrifuging 10× *g* of the yogurt sample at 1600 rpm, for 10 min. After centrifugation, the supernatant was decanted and weighed on an analytical laboratory balance [40]. This parameter was determined on the 1st, 7th, 14th, 17th and 20th days.

### 2.7. Mathematical Modeling

The MATLAB program (MathWorks, Inc., Natick, MA, USA) was applied for the information analysis of the experimental data, which allowed us to establish the influences between the different quantities measured during the tests. Names of textural parameters and general acceptability are introduced in the rectangles of the graph, and the values of mutual information, measured in bits, are indicated on the arrows of the graph. The more pronounced the influence of concentration of AP powder and storage period on textural parameters and overall acceptability, the higher the value of the bit [41].

### 2.8. Statistical Analysis

All calculations were performed using Microsoft Office Excel 2007 (Microsoft, Redmond, WA, USA). Data obtained in this study are presented as mean values ± the standard error of the mean, calculated from three parallel experiments. The comparison of average values was based on the one-way analysis of variance (ANOVA), according to Tukey’s test, at a significance level of *p* ≤ 0.05, using the Staturphics program, Centurion XVI 16.1.17 (Statgraphics Technologies, Inc., The Plains, VA, USA).

## 3. Results

### 3.1. Apple Pomace Powder Characterization

#### 3.1.1. Physicochemical Analysis of the Apple Pomace Powder

The physicochemical parameters of the AP powder are presented in Table 2.

The fat and ash content in the AP was 3.03% and 1.67%, respectively. These values are in accordance with the results obtained by other authors [42,43,44,45], who determined that the ash content of AP was 2% and that the fat content varied from 2.20 to 4.40 g/100 g pomace—the values were influenced by the fat content of apple seeds.

The protein content of AP was 5.27%. Antonic et al. (2020) [46] showed that the protein content in AP varied from 1.2 to 6.91 g/100 g depending on the apple variety, the AP processing method and the protein dosage method. Similar results were also obtained by Rana et al. (2015) [47], who analyzed the chemical composition of 11 varieties of apples, the protein content varied from 3.75 to 4.65 g/100 g, depending on the variety.

The total dietary fibers content in AP was 62.73%. The result of the determination of the total dietary fibers in AP, as reported by other authors, varies from 51.1% [44] to 74.0% [47]. Islam et al. (2020) [48] reported that the pomace obtained from organic cranberries contains, on average, 46.3% of total dietary fibers and 15.5% of insoluble dietary fibers, respectively.

The pectin content of the analyzed AP (extracted with citric acid solution pH~2, at 90 °C, for 130 min) was 23.12%. According to the research results of Ma et al. (2019) [49], the pectin content of AP, extracted with 10% (*w/w)* acetic acid solution, at 100 °C, was 19.6%. Significant differences between the chemical composition of AP (content of lipids, proteins, polyphenols, dietary fibers, and the ratio between soluble and insoluble dietary fibers) are determined by the apple variety, cultivation and processing conditions of AP [44,45,50]. The total dietary fiber content, including the insoluble dietary fiber in AP, is higher compared to berries and whole grains. In addition, AP dietary fibers are characterized by a more optimal ratio between the soluble and insoluble fraction and the presence of a higher concentration of bioactive compounds [6].

#### 3.1.2. Antioxidant Compounds and Antioxidant Activity in Apple Pomace Powder

AP is also an important source of antioxidants such as polyphenols, including flavonoids. Polyphenols influence the sensory properties of foods (taste, color, aroma, astringency, etc.), and also the oxidative stability of foods—being a reducing agent, they protect living cells from the action of free radicals. Table 3 shows the content of TPC, TFC, tannins, carotenoids and AA in AP powder.

The TPC and TFC in the AP was 728.8 mg GAE/100 g DW and 246.5 mg QE/100 g DW, respectively. The TPC and TFC values in the AP are in agreement with the results presented by Gorjanović et al. (2020) [51], who determined the content of TPC and TFC in samples of dried industrial pomace from different apple varieties and found that the average TPC was 7.7 mg GAE/g DW and TFC 24.8 mg QE/g DW. The AP of the Idared variety of apples recorded the highest TPC—of 8.1 mg GAE/g—and TFC—of 34.6 mg QE/g. The extraction of polyphenols was carried out in a 50% (*v/v*) hydroethanolic solution, at room temperature, for 60 min. Rana et al. (2015) [47] recorded a TPC of 2.17 mg GAE/g DW and a TFC of 0.45 mg QE/g DW, which was calculated for the pomace extract in 50% (*v/v*) hydroethanolic solution, at a temperature of 60 °C, for 30 min.

The tannin content in the studied AP was 63.54 mg TAE/100 g DW. According to research by Krasnova and Segliòa (2019) [52], the tannin content varied depending on the apple variety, from 29.11 to 73.40 mg TAE/100 g DW.

The total carotenoid content in the analyzed AP was 4.93 mg/100 g DW. According to the studies carried out by Radenkovs et al. (2018) [53], the carotenoid content in the oil recovered from AP was 14.5 and 5.1 mg/mL. The low content of carotenoids in the AP was probably determined by the low number of seeds. Polyphenols—as well as other bioactive compounds, such as vitamins, carotenoids, minerals, etc.—influenced the AA of the food product [43]. In addition, the AA can be influenced by the content of pectin in the AP [54].

The AA of the AP was 2433 µmol TE/100 g DW. According to Candrawinata et al. (2014) [55], the AA in the AP was 500.21 μg TE/100 g DW. Increasing the extraction temperature to 70 °C led to an increase in AA of up to 912.24 μg TE/100 g DW. The highest AA was determined for AP extract in methanol, which was 2038.53 μg TE/100 g DW. Gorjanović et al. (2020) [51] showed an AA between 2.2 and 4.5 mmol TEA/100 g of dry AP. The incorporation of AP into food formulas improved the AA by preventing or delaying oxidative reactions, i.e., the oxidation of lipids in the food matrix [56]. Therefore, the bioactive components from AP can be harnessed by using it in the formulation of functional yogurt.

In the next stages of this study, yogurt with the addition of AP powder was analyzed to verify the hypothesis that this type of addition could be regarded as a natural stabilizer, with antioxidant activity.

### 3.2. Physicochemical, Color and Antioxidant Analysis of Yogurt with Apple Pomace Powder

The physicochemical parameters, color and the AA of the yogurt with AP powder are presented in Table 4.

The total solids content of the yogurt varied between 14.40%, in the case of the control yogurt, and 15.04%, in the case of the yogurt with 1% AP. Increasing the added AP content caused a non-essential increase in the fat content from 0.15% (Y) to 0.18% (1.0%YAP).

Regarding the total dietary fiber content—including pectic substances—the addition of 0.4% of AP increased the dietary fiber content to 0.25%, while the addition of 1% of AP increased the dietary fiber content to 0.63%. It has been shown that, at a low pH (≤4.5), pectin is absorbed by electrostatic interactions on the surface of the casein micelle, in areas rich in carboxylic groups [57]. In addition, the formation of an established yogurt matrix occurs due to anionic interactions with calcium ions (Ca^2+^), which promote the interpenetration of hydrated pectin chains in the protein network [58]. AP, in a proportion of 1%, conditioned the increase in the level of insoluble dietary fiber in yogurt by up to 0.14%. Insoluble dietary fibers from yogurt, distributed in the serum phase of the casein network, hydrate and swell, contributing significantly to the retention and immobilization of the whey, and the formation of a connected colloidal network [40]. Both interactions suggest that AP acts as an active filler, with the additional stabilization of the serum phase in the well-developed casein network [58].

Similarly, increasing the content of AP in yogurt resulted in an increase in the AA. In the case of yogurt with 1.0% AP, the AA reached the value of 29.8 μmol TE/100 g sample. Even the smallest amount of AP (0.2%) caused an increase in the AA by approximately 0.3 times, compared to the control sample and a 1% amount of AP—56 times. These findings are in agreement with those of Du et al. (2022) [59] and Ivanova et al. (2021) [60], who reported increased an AA in yogurt that was produced with mulberry pomace and extract from strawberry pomace, respectively.

The color of the yogurt is an important indicator, which influences the quality, commercial value and acceptability of the product, and is directly influenced by the type and concentration of the additive used in the recipe [61]. As the amount of AP in the yogurt samples increased, the lightness (L*) values of the samples decreased from 77.27 (Y) to 73.36 (1.0%YAP). The values of the red–green component (a*) were located in the negative domain, which confirmed that the green tone predominated over the red in all the samples. In the case of the yellow–blue component (b*), in all the yogurt samples the yellow color prevailed due to the addition of AP. The total color difference (ΔE*) of the yogurt samples with added AP increased from 0.77 (0.2%YAP) to 4.5 (1.0%YAP). According to Ramirez-Rodriguez et al. (2011) [62], consumers can perceive a change in the color of a food product when the ΔE* value is above three, therefore, the yogurt samples with added AP above 0.6% showed a notable color difference.

### 3.3. Evolution of the Yogurt with Apple Pomace Powder Characteristics during Storage

#### 3.3.1. Sensory Analysis

The sensory quality of yogurt, after manufacturing and during storage, is one of the determining factors in consumer choice. In this study, to evaluate the acceptability of the yogurt with AP, a sensory analysis was performed. The results of the sensory analysis of the yogurt with AP powder, during storage, are presented in the Table 5.

High scores for sensory properties were assigned to all the samples analyzed by the panel of assessors. The 0.6%YAP and 0.8%YAP samples were evaluated with the highest score, as they were characterized by a clot of a firm, creamy consistency, and a specific odor and taste of yogurt and apple. It is worth noting that a content higher than 0.8% AP, created a coarse sensation of AP powder in the oral cavity, which also determined the decrease in the overall acceptability of the 1.0%YAP sample. However, the addition of sugar, flavorings and fruit fillers in the yogurt recipe with 1% AP could improve the sensory quality of the yogurt.

During the storage period, in general, the sensory quality of the yogurt samples essentially did not change, except for the 1.0%YAP sample. On the 20th day of storage, the yogurt samples, 0.6%YAP and 0.8%YAP, were appreciated with the highest score. Similar results were reported by Marchiani et al. (2016) [63], whereby the overall acceptability scores of the grape pomace yogurt were higher than the scores of a control yogurt. Jovanovic´ et al. (2021) [64] also demonstrated an acceptable palatability of yogurt, with the addition of granulated beetroot pomace flour.

#### 3.3.2. pH Evolution

The pH value evolution of the yogurt with AP powder during the fermentation and storage is presented in Table 6.

According to the data presented in Table 6, the evolution of the pH value during the fermentation and storage period was determined by the development of lactic bacteria from the starter culture, as well as by the addition of AP to the milk. The end of the yogurt’s fermentation period was attested at the pH value~4.6 [65]. The fermentation time of the yogurt samples, generally, varied between 7 and 8 h. Increasing the amount of AP led to a reduction in the fermentation time. Thus, for the 0.4%YAP and 0.6%YAP samples, the fermentation time was reduced from 8 h (Y) to 7.5 h, and in the case of the 0.8%YAP and 1.0%YAP samples, to 7 h. At an addition of 0.2% AP, no such effect was observed. The decrease in the fermentation time of the yogurt samples with the addition of AP over 0.4%, was probably determined by the acidic nature of the AP, due to the presence of organic acids [6]. The results obtained are consistent with those reported by Wang et al. (2019) [66].

Throughout the storage period (20 days), the pH of all the yogurt samples decreased gradually, and the lowest values were recorded on the last day. The slow co-fermentation of the lactic acid bacteria from the starter culture, especially *Streptococcus thermophilus* and *Lactobacillus bulgaricus*, during storage caused the continuous acidification of the yogurt. The polysaccharides in AP could serve as prebiotics for lactic acid bacteria [67], causing additional lactic acid production. The changes in the pH of the investigated samples during storage were consistent with the results of the studies reported by other authors [39,58,59].

#### 3.3.3. Texture Profile Analysis

Knowledge of the rheological properties of yogurts is important from a technological point of view. Due to their complex microstructure, yogurt gels are susceptible to temperature, shear forces and storage time [14]. The structure and texture of yogurt is formed mainly in the milk fermentation process, but also as a result of the addition of hydrocolloids [17]. The rheological and textural properties of fermented dairy products depend on their structural arrangement and the microstructure of the protein network [67]. The TPA parameters’ (firmness, springiness, cohesiveness, adhesiveness and gumminess) evolution of the yogurt with AP powder during storage are indicated in Table 7.

The results in Table 7 attest that AP significantly affected the textural properties of yogurt. As the amount of AP increased from 0.2 to 1%, the firmness values increased from 1297.3 g to 1944.5 g. The springiness of the yogurt increased with the increase in the AP levels. The maximum springiness of the yogurt was recorded at AP levels of 0.8 and 1.0% (1.375% and 1.401%, respectively).

Cohesiveness is correlated with the consumer acceptability of yogurt and is an important parameter for yogurt texture analysis. The evolution of the cohesiveness values demonstrated a direct correlation between the AP content and the cohesion of the yogurt. The maximum value of cohesiveness was recorded in the case of the 1.0%YAP sample (0.703%). The tendency to increase cohesiveness in yogurt samples could be due to the strength of the yogurt’s structure conferred by AP.

The adhesiveness of the yogurts with AP showed a decreasing trend, from 1306.9 g∙s (0.2%YAP) to 1219.1 g∙s (1.0%YAP). The consumer acceptability of yogurt is considered to increase inversely with the value of yogurt stickiness [68].

Gumminess is another important parameter for the textural analysis of yogurt [69]. Similar to adhesiveness, increasing the concentration of AP led to a decrease in the gumminess values of the yogurt samples, from 0.874% (0.2%YAP) to 0.382% (1.0%YAP).

An improved texture has also been reported for yogurts with incorporated by-products, such as blueberry pomace (4.5%) [40], passion fruit peel powder (0.5% and 1.0%) [70] and carrot cell wall particles (1% and 2%) [71]. However, the opposite results were also found by Tseng et al. (2013) [72], who demonstrated that yogurt with 3% wine grape pomace (added after fermentation) had a lower viscosity than yogurt without this addition.

The results of the texture analysis, during 20 days of storage, suggested that AP supplementation led to a stable system and the formation of a strong three-dimensional network in the yogurt. Therefore, the AP caused the degree of crosslinking in the gel network to increase and, as a result, a firmer gel structure was formed.

During the 20 days of storage, all the yoghurt samples with added AP showed an increase in the values of firmness, elasticity and cohesion, and a decrease in the values of adhesiveness and gumminess, which proves that the addition of AP led to the formation of a strong three-dimensional network in the yogurt, which was stable during storage. Such an effect could be attributed to the gelling ability of pectin and other soluble dietary fibers released from AP in milk, as well as the strengthening of the gel structure by the insoluble pomace particles [66].

#### 3.3.4. Syneresis

Table 8 shows the syneresis evolution of yogurt with AP powder during storage, which confirms the TPA results.

The highest syneresis value was recorded for sample Y, 26.65%. This fact can be explained due to the inability of the yogurt gel to retain the serum phase, as a result of the over-time weakening of the casein network, which led to the release of the whey [73]. The addition of AP in the yogurt’s structure reduced syneresis from 25.72% (0.2%YAP) to 24.38% (1.0%YAP). Thus, the addition of 1% AP to the yogurt led to the most effective whey retention. In accordance with the results obtained in this research, the yogurt samples that were fortified with 4.5% cranberry pomace did not show whey removal [40]. Furthermore, Damirkol and Taraksi (2018) [74] claimed that syneresis occurs only in yogurt samples supplemented with grape (*Vitis labrusca* L.) pomace.

During storage, the syneresis of the control sample showed an increasing trend, while the syneresis of the yogurt samples with AP gradually decreased during the 20 days of storage. Increasing the amount of AP and total solids, respectively, led to the strengthening of the lactic gel network and to the reduction in yogurt syneresis. The decrease in the syneresis of yogurts with AP can be attributed to the interactions between the milk and the dietary fiber, especially pectin [75]. Moreover, the modification of syneresis may be related to polyphenol–protein interactions since polyphenols have a high affinity for casein. Covalent or non-covalent interactions between polyphenols and casein lead to the formation of stable soluble complexes [76].

In general, in this study, the texture parameters of the yogurt samples correlated with their sensory properties, except for the 1.0%YAP sample. This sample demonstrated high texture characteristics, however, from a sensory point of view, it was rated with a low score as a result of the coarse sensation of the AP powder in the oral cavity. The addition of AP in proportions of 0.6 and 0.8%, created a yogurt with a firm texture, high elasticity and cohesiveness, reduced gumminess and stickiness, and with high overall acceptability, which suggests that this is the optimal dosage for industrial production.

### 3.4. Mathematical Modeling

**Figure 1:**
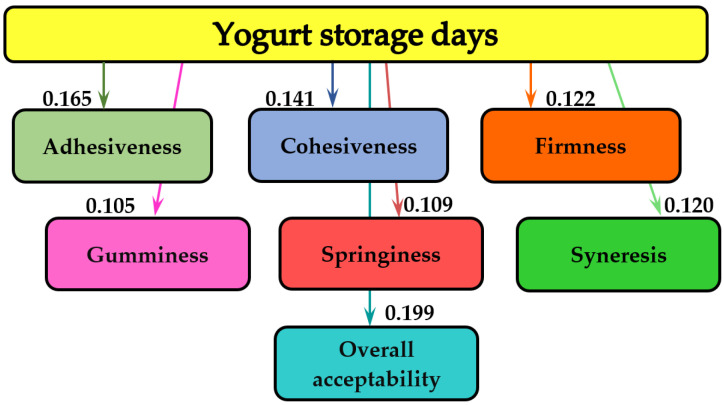
The informational analysis of the influence of storage days on textural parameters and overall acceptability of yogurt.

The influence of the storage period and the concentrations of AP added to the yogurt on the texture parameters and the sensory analysis—especially the overall acceptability of the obtained samples—were evaluated. The analysis of mutual information was applied to evaluate the measure of the influence of the storage period and of the AP concentrations on the texture characteristics and on the overall acceptability of the yogurt samples. This mathematical modeling method has also been applied in studying the influence of physicochemical quality indicators on the texture characteristics of dry-aged beef, for 35 days [77]. The influence of measuring the various quantities of sea buckthorn flour and rosehip powder on the quality of wheat bread and gingerbread has also been evaluated by applying mutual analysis [30,78]. Figure 1 shows the mutual analysis of the influence of storage days on the textural parameters (adhesiveness, cohesiveness, firmness, gumminess, springiness and syneresis) and on the overall acceptability of the yogurt in this study. The mutual influence values are shown on the arrows.

As shown in Figure 1, the storage period of the yogurt samples with different concentrations of added AP did not essentially influence the texture parameters or the overall acceptability. The values of the informational analysis for the texture characteristics ranged from 0.105 bits (gumminess) to 0.165 bits (adhesiveness), and, for overall acceptability, it was 0.199 bits.

**Figure 2:**
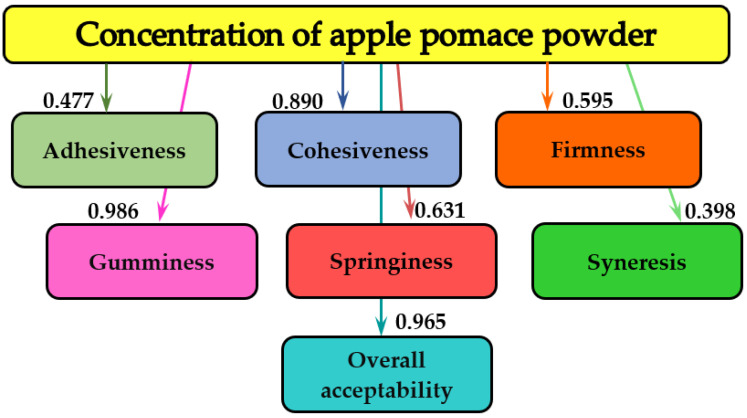
The informational analysis for the influence of concentration of AP powder on textural parameters and overall acceptability of yogurt.

Figure 2 summarizes the effects of the different amounts of AP powder that were added to the yogurt samples on the textural parameters and on the overall acceptability.

Figure 2 shows that the concentration of the AP added to the samples essentially influenced the textural parameters and the sensory quality of the yogurt. In the case of the textural characteristics, the gumminess was the most influenced (0.986 bits), followed by the cohesiveness (0.890 bits) and the springiness (0.631 bits). For syneresis, the information analysis value was the lowest (0.398 bits). The amount of AP added to the yogurt largely influenced the quality of the yogurt samples, because the information analysis value for the overall acceptability was 0.965 bits.

## 4. Conclusions

The physicochemical composition of the AP obtained after extracting the juice from the Golden Delicious apple variety was determined. The AP was rich in proteins—5.27%, and dietary fibers—62.73%, including pectin—23.12%.

The content of antioxidant compounds in AP was analyzed, as follows: total polyphenols (728.8 mg GAE/100 g DW); flavonoids (246.5 mg QE/100 g DW); tannins (63.54 mg TAE/100 g DW); carotenoids (4.93 mg/100 g DW) and the antioxidant activity (2433 µmol TE/100 g DW). The obtained results demonstrated that AP powder could be regarded as a natural stabilizer, with antioxidant properties.

The physicochemical composition, color and antioxidant activity of the yogurt samples with AP powder (0.2–1.0%) were determined in relation to the control sample. The increase in the total content of dry substances—from 14.40%, in the case of the control yogurt, to 15.04%, in the case of the yogurt with 1% AP; in the content of dietary fibers—up to 0.63%; and in the content of insoluble dietary fibers—up to 0.14%—led to the formation of a stable yogurt matrix. A significant increase in the antioxidant activity, correlated to the AP content, was attested. The color of the yogurt showed that the green tone prevailed over the red in all the analyzed samples. Notable color differences were attested in the yogurt samples with the addition of AP exceeding 0.6%.

The influence of AP addition on the yogurt fermentation process was determined. In the case of the 0.8%YAP and 1.0%YAP samples, the fermentation time was reduced by 1 h, which was probably determined by the presence of organic acids in the AP.

The sensory characteristics of the yogurt samples with AP during storage (1–20 days) were evaluated. The 0.6%YAP and 0.8%YAP samples were evaluated with the highest score, as they were characterized by a clot of a firm, creamy consistency, and a specific odor and taste of yogurt and apple.

The addition of AP significantly influenced the textural properties of the yogurt during storage (20 days), with the firmness values varying from 1390.3 g (control sample) to 2244.5 g (1%YAP). The addition of AP led to the formation of a strong and stable three-dimensional network in the yogurt, during storage, a fact attributed to the gelling capacity of pectin and the other soluble dietary fibers, as well as to the strengthening of the gel structure as a result of the swelling of the insoluble dietary fibers. This was also confirmed by the significant reduction in syneresis in the yogurt samples with added AP, during the 20 days of storage, from 28.13% (control sample) to 22.18% (1%YAP).

The influence of the AP additions and the storage period on the textural characteristics and on the overall acceptability of the yogurt samples was analyzed by the mutual information method. It was found that the rate of AP greatly influenced the quality of the yogurt, with the information analysis value for global acceptability being 0.965 bits.

## Figures and Tables

**Table 1:** Sensory characteristics of the yogurt.

Sensory Characteristics	Description
Appearance and consistency	The curd is fine, homogeneous, has a fluid consistency, without gas bubbles. A slight removal of whey, to a maximum of 2%, is allowed.
Color	White, yellowish-white or specific to the ingredients.
Odor	Specific to the type, with lactic fermentation characteristics, pleasant. With the odor of ingredients used in the case of those with added fruit. No foreign odor was allowed.
Taste	Specific to the type, with lactic fermentation characters, pleasant and sour. With the taste of ingredients used in the case of those with added fruit. No foreign taste was allowed.

**Table 2:** Physicochemical parameters of the apple pomace powder.

Parameters	Value
Moisture, % Titrabile acidity, % expressed in malic acid Soluble solids content, ^o^Brix Fat content, % Protein content, % Total dietary fibers content, % Pectin content, % Insoluble dietary fibers content, % Ash content, %	7.84 ± 0.05 0.22 ± 0.01 15.82 ± 0.01 3.03 ± 0.18 5.27 ± 0.09 62.73 ± 1.46 23.12 ± 1.70 14.05 ± 0.44 1.67 ± 0.02

Values in the table represent means of three replicated trials, ± standard deviation.

**Table 3:** Content of biologically active compounds and antioxidant activity in apple pomace powder.

Compounds	Value
Total polyphenol content, mg GAE/100 g DW Total flavonoid content, mg QE/100 g DW Total tannins, mg TAE/100 g DW Total carotenoids, mg/100 g DW Antioxidant activity (DPPH), µmol TE/100 g DW	728.8 ± 25.5 246.5 ± 31.2 63.54 ± 5.71 4.93 ± 0.27 2433 ± 44.3

Values in the table represent means of three replicated trials, ± standard deviation.

**Table 4:** Physicochemical, color and antioxidant activity of yogurt with apple pomace powder.

Values	Samples					
	Y	0.2%YAP	0.4%YAP	0.6%YAP	0.8%YAP	1.0%YAP
Total solids content, %	14.40 ± 0.01 ^a^	14.55 ± 0.02 ^b^	14.69 ± 0.01 ^c^	14.82 ± 0.02 ^d^	14.89 ± 0.01 ^d^	15.04 ± 0.02 ^e^
Fat content, %	0.15 ± 0.002 ^a^	0.15 ± 0.001 ^a^	0.16 ± 0.002 ^b^	0.17 ± 0.001 ^c^	0.17 ± 0.001 ^c^	0.18 ± 0.002 ^d^
Total dietary fibers content, %	n.d.	0.13 ± 0.01 ^a^	0.25 ± 0.02 ^b^	0.38 ± 0.02 ^c^	0.50 ± 0.0 ^d^	0.63 ± 0.03 ^e^
Insoluble dietary fiber content, %	n.d.	0.03 ± 0.01 ^a^	0.06 ± 0.01 ^b,c^	0.08 ± 0.01 ^c^	0.11 ± 0.02 ^d,e^	0.14 ± 0.01 ^e^
Luminance (L*)	77.27 ± 0.10 ^d^	76.79 ± 0.04 ^d^	74.87 ± 0.02 ^c^	74.37 ± 0.08 ^c^	73.9 ± 0.02 ^b^	73.36 ± 0.05 ^a^
Red/green component (a*)	−2.56 ± 0.04 ^a^	−1.96 ± 0.02 ^b^	−1.57 ± 0.02 ^c^	−1.5 ± 0.03 ^c,d^	−1.3 ± 0.06 ^d,e^	−1.25 ± 0.03 ^e^
Yellow/blue component (b*)	6.27 ± 0.03 ^a^	6.35 ± 0.09 ^a,b^	6.59 ± 0.05 ^b^	7.56 ± 0.04 ^c^	7.5 ± 0.01 ^c^	8.06 ± 0.04 ^d^
Total color differences (ΔE*)	-	0.77 ± 0.05 ^a^	2.62 ± 0.12 ^b^	3.35 ± 0.07 ^c^	3.80 ± 0.08 ^d^	4.50 ± 0.04 ^e^
Antioxidat Activitity (DPPH), μmol TE/100 g	0.52 ± 0.03 ^a^	0.67 ± 0.07 ^a^	1.75 ± 0.14 ^a^	7.15 ± 0.83 ^b^	11.01 ± 1.96 ^c^	29.80 ± 0.79 ^d^

-, ΔE* was calculated relative to the control sample (Y). n.d., not determined. Values in the table represent means of three replicated trials, ± standard deviation. Different letters (^a–e^) designate statistically different results (*p* ≤ 0.05).

**Table 5:** Sensory properties (score) and evolution of yogurt with apple pomace powder, during storage.

Sensory Properties	Storage Period	Samples					
		Y	0.2%YAP	0.4%YAP	0.6%YAP	0.8%YAP	1.0%YAP
Appearance and consistency	1 7 14 17 20	4.05 ± 0.04 ^f^ 4.05 ± 0.03 ^f^ 4.00 ± 0.05 ^e,f^ 3.96 ± 0.04 ^e^ 3.82 ± 0.01 ^d^	4.23 ± 0.06 ^h,i^ 4.23 ± 0.05 ^h,i^ 4.20 ± 0.07 ^g,h^ 4.20 ± 0.05 ^g,h^ 4.12 ± 0.04 ^f,g^	4.41 ± 0.02 ^j^ 4.41 ± 0.05 ^j^ 4.52 ± 0.04 ^k,l^ 4.54 ± 0.03 ^k,l^ 4.60 ± 0.03 ^l^	4.41 ± 0.03 ^j^ 4.41 ± 0.02 ^j^ 4.52 ± 0.04 ^k,l^ 4.54 ± 0.02 ^k,l^ 4.60 ± 0.03 ^l^	4.83 ± 0.05 ^n,o^ 4.83 ± 0.03 ^n,o^ 4.89 ± 0.04 ^o,p^ 4.92 ± 0.02 ^o,p^ 4.90 ± 0.01 ^o^	3.48 ± 0.04 ^b,c^ 3.48 ± 0.05 ^b,c^ 3.40 ± 0.06 ^b^ 3.38 ± 0.01 ^b^ 3.26 ± 0.02 ^a^
Color	1 7 14 17 20	5.00 ± 0.00 ^p^ 5.00 ± 0.00 ^p^ 5.00 ± 0.00 ^p^ 5.00 ± 0.00 ^p^ 5.00 ± 0.00 ^p^	5.00 ± 0.00 ^p^ 5.00 ± 0.00 ^p^ 5.00 ± 0.00 ^p^ 5.00 ± 0.00 ^p^ 5.00 ± 0.00 ^p^	5.00 ± 0.00 ^p^ 5.00 ± 0.00 ^p^ 5.00 ± 0.00 ^p^ 5.00 ± 0.00 ^p^ 5.00 ± 0.00 ^p^	5.00 ± 0.00 ^p^ 5.00 ± 0.00 ^p^ 5.00 ± 0.00 ^p^ 5.00 ± 0.00 ^p^ 5.00 ± 0.00 ^p^	5.00 ± 0.00 ^p^ 5.00 ± 0.00 ^p^ 5.00 ± 0.00 ^p^ 5.00 ± 0.00 ^p^ 5.00 ± 0.00 ^p^	4.22 ± 0.03 ^g,h^ 4.21 ± 0.02 ^g,h^ 4.21 ± 0.05 ^g,h^ 4.20 ± 0.04 ^g,h^ 4.20 ± 0.04 ^g,h^
Odor	1 7 14 17 20	5.00 ± 0.00 ^p^ 5.00 ± 0.00 ^p^ 4.62 ± 0.02 ^l^ 4.60 ± 0.03 ^l^ 4.56 ± 0.04 ^k,l^	5.00 ± 0.00 ^p^ 5.00 ± 0.00 ^p^ 4.88 ± 0.03 ^o^ 4.84 ± 0.01 ^o^ 4.84 ± 0.01 ^o^	5.00 ± 0.00 ^p^ 5.00 ± 0.00 ^p^ 4.92 ± 0.04 ^o,p^ 4.88 ± 0.02 ^o^ 4.86 ± 0.03 ^o^	5.00 ± 0.00 ^p^ 5.00 ± 0.00 ^p^ 4.80 ± 0.02 ^n^ 4.80 ± 0.01 ^n^ 4.78 ± 0.03 ^n^	5.00 ± 0.00 ^p^ 5.00 ± 0.00 ^p^ 4.80 ± 0.01 ^n^ 4.78 ± 0.02 ^n^ 4.72 ± 0.01 ^m^	4.64 ± 0.02 ^l,m^ 4.58 ± 0.02 ^l^ 4.48 ± 0.03 ^j,k^ 4.48 ± 0.04 ^j,k^ 4.24 ± 0.03 ^h^
Taste	1 7 14 17 20	5.00 ± 0.00 ^p^ 5.00 ± 0.00 ^p^ 4.80 ± 0.02 ^n^ 4.60 ± 0.04 ^l^ 4.56 ± 0.03 ^k,l^	5.00 ± 0.00 ^p^ 5.00 ± 0.00 ^p^ 4.86 ± 0.03 ^o^ 4.84 ± 0.02 ^n,o^ 4.76 ± 0.04 ^m,n^	5.00 ± 0.00 ^p^ 5.00 ± 0.00 ^p^ 4.92 ± 0.04 ^o,p^ 4.88 ± 0.03 ^o^ 4.84 ± 0.04 ^n,o^	5.00 ± 0.00 ^p^ 5.00 ± 0.00 ^p^ 4.90 ± 0.03 ^o,p^ 4.80 ± 0.02 ^n^ 4.64 ± 0.02 ^l,m^	5.00 ± 0.00 ^p^ 5.00 ± 0.00 ^p^ 4.88 ± 0.03 ^o^ 4.78 ± 0.04 ^n^ 4.68 ± 0.03 ^m^	4.57 ± 0.04 ^k,l^ 4.42 ± 0.02 ^j^ 4.36 ± 0.03 ^i,j^ 4.20 ± 0.04 ^g,h^ 4.00 ± 0.02 ^e,f^
Overall acceptability	1 7 14 17 20	4.76 ± 0.04 ^m,n^ 4.76 ± 0.03 ^m,n^ 4.61 ± 0.02 ^l^ 4.54 ± 0.02 ^k,l^ 4.49 ± 0.03 ^j,k^	4.81 ± 0.02 ^n,o^ 4.81 ± 0.01 ^n,o^ 4.74 ± 0.02 ^m,n^ 4.72 ± 0.01 ^m^ 4.68 ± 0.02 ^m^	4.85 ± 0.02 ^o^ 4.85 ± 0.01 ^o^ 4.84 ± 0.02 ^n,o^ 4.83 ± 0.02 ^n,o^ 4.83 ± 0.01 ^n,o^	4.96 ± 0.01 ^p^ 4.93 ± 0.02 ^o,p^ 4.90 ± 0.02 ^o,p^ 4.88 ± 0.01 ^o^ 4.82 ± 0.02 ^n,o^	4.96 ± 0.01 ^p^ 4.96 ± 0.01 ^p^ 4.89 ± 0.02 ^o^ 4.87 ± 0.02 ^o^ 4.83 ± 0.01 ^n,o^	4.23 ± 0.02 ^h^ 4.18 ± 0.01 ^g^ 4.11 ± 0.01 ^g^ 4.07 ± 0.02 ^e^ 3.93 ± 0.01 ^e^

Values in the table represent the means of three replicated trials, ± standard deviation. Different letters (^a–p^) designate statistically different results (*p* ≤ 0.05).

**Table 6:** pH value evolution of yogurt with apple pomace powder during fermentation and storage.

Parameter	Times	Samples					
		Y	0.2%YAP	0.4%YAP	0.6%YAP	0.8%YAP	1.0%YAP
Fermentation							
pH	0 h 2 h 4 h 6 h 7 h 7.5 h 8 h	6.58 ± 0.02 ^n^ 6.42 ± 0.01 ^m^ 6.04 ± 0.02 ^k^ 5.16 ± 0.02 ^g^ 4.95 ± 0.03 ^f^ 4.85 ± 0.01 ^e^ 4.62 ± 0.02 ^d^	6.58 ± 0.01 ^n^ 6.42 ± 0.02 ^m^ 5.92 ± 0.01 ^j^ 5.02 ± 0.01 ^f^ 4.91 ± 0.03 ^f^ 4.82 ± 0.02 ^e^ 4.60 ± 0.04 ^c,d^	6.58 ± 0.02 ^n^ 6.38 ± 0.01 ^m^ 5.85 ± 0.02 ^i,j^ 4.92 ± 0.04 ^e,f^ 4.76 ± 0.01 ^e^ 4.62 ± 0.03 ^d^ n.d.	6.58 ± 0.03 ^n^ 6.38 ± 0.02 ^m^ 5.85 ± 0.03 ^i,j^ 4.92 ± 0.01 ^f^ 4.76 ± 0.03 ^d,e^ 4.61 ± 0.01 ^d^ n.d.	6.58 ± 0.01 ^n^ 6.38 ± 0.04 ^m^ 5.75 ± 0.01 ^i^ 4.86 ± 0.02 ^e^ 4.62 ± 0.02 ^d^ n.d. n.d.	6.58 ± 0.02 ^n^ 6.20 ± 0.03 ^l^ 5.55 ± 0.01 ^h^ 4.85 ± 0.03 ^e^ 4.60 ± 0.04 ^c,d^ n.d. n.d.
Storage							
pH	1 day 7 day 14 day 17 day 20 day	4.60 ± 0.02 ^d^ 4.58 ± 0.01 ^c,d^ 4.57 ± 0.03 ^c,d^ 4.50 ± 0.02 ^c^ 4.47 ± 0.03 ^c^	4.59 ± 0.05 ^d^ 4.57 ± 0.03 ^c,d^ 4.55 ± 0.02 ^c^ 4.50 ± 0.04 ^c^ 4.45 ± 0.01 ^c^	4.60 ± 0.02 ^d^ 4.56 ± 0.01 ^c^ 4.50 ± 0.03 ^c^ 4.47 ± 0.01 ^c^ 4.41 ± 0.02 ^b,c^	4.59 ± 0.01 ^d^ 4.52 ± 0.03 ^c^ 4.48 ± 0.02 ^c^ 4.42 ± 0.05 ^b,c^ 4.39 ± 0.01 ^b^	4.59 ± 0.03 ^c,d^ 4.46 ± 0.02 ^c^ 4.38 ± 0.03 ^b^ 4.35 ± 0.01 ^b^ 4.26 ± 0.04 ^a,b^	4.58 ± 0.01 ^c,d^ 4.42 ± 0.02 ^b,c^ 4.35 ± 0.02 ^b^ 4.30 ± 0.04 ^b^ 4.24 ± 0.03 ^a,b^

n.d.—not determined because the sample was placed in storage. Values in the table represent the means of three replicated trials, ± standard deviation. Different letters (^a–n^) designate statistically different results (*p* ≤ 0.05).

**Table 7:** Texture parameters’ evolution of yogurt with apple pomace powder during storage.

Texture Parameters	Storage Period, Days	Samples					
		Y	0.2%YAP	0.4%YAP	0.6%YAP	0.8%YAP	1.0%YAP
Firmness, g	1 7 14 17 20	1235.0 ± 29.2 ^a^ 1271.8 ± 42.4 ^a,b^ 1316.5 ± 25.5 ^a,b^ 1377.2 ± 31.8 ^b^ 1390.3 ± 41.7 ^b,c^	1297.3 ± 26.8 ^a,b^ 1307.6 ± 41.9 ^a,b^ 1321.1 ± 36.5 ^a,b^ 1329.3 ± 28.6 ^b^ 1339.0 ± 36.8 ^b^	1343.6 ± 31.5 ^b^ 1351.9 ± 42.9 ^b^ 1457.9 ± 53.9 ^c^ 1562.9 ± 48.4 ^d^ 1574.1 ± 38.1 ^d^	1442.2 ± 29.6 ^c^ 1483.4 ± 35.3 ^c,d^ 1646.4 ± 42.8 ^e^ 1736.5 ± 51.8 ^e,f^ 1804.9 ± 58.5 ^f,g^	1661.8 ± 46.7 ^e,f^ 1751.4 ± 49.1 ^e,f^ 2064.7 ± 41.3 ^i,j^ 2105.7 ± 49.6 ^i,j^ 2185.5 ± 43.7 ^j,k^	1944.5 ± 36.8 ^h^ 1992.4 ± 41.9 ^h,i^ 2191.2 ± 39.7 ^j,k^ 2269.9 ± 29.8 ^k^ 2244.5 ± 32.5 ^k^
Springiness, %	1 7 14 17 20	1.001 ± 0.001 ^a^ 1.004 ± 0.003 ^a^ 1.075 ± 0.005 ^a^ 1.093 ± 0.006 ^a^ 1.098 ± 0.002 ^a^	1.290 ± 0.005 ^b^ 1.319 ± 0.006 ^b^ 1.339 ± 0.008 ^b^ 1.391 ± 0.007 ^c^ 1.403 ± 0.011 ^c,d^	1.303 ± 0.008 ^b,c^ 1.300 ± 0.006 ^b,c^ 1.410 ± 0.012 ^c,d^ 1.431 ± 0.017 ^c^ 1.447 ± 0.021 ^c^	1.329 ± 0.012 ^b,c^ 1.391 ± 0.008 ^c^ 1.620 ± 0.019 ^e^ 1.920 ± 0.020 ^f^ 2.080 ± 0.027 ^g,h^	1.375 ± 0.009 ^c^ 1.495 ± 0.005 ^d^ 1.983 ± 0.018 ^f,g^ 2.148 ± 0.021 ^h^ 2.218 ± 0.027 ^h,i^	1.401 ± 0.011 ^c,d^ 1.607 ± 0.007 ^e^ 2.064 ± 0.016 ^g^ 2.182 ± 0.027 ^h^ 2.269 ± 0.030 ^i^
Cohesiveness, %	1 7 14 17 20	0.278 ± 0.001 ^a^ 0.280 ± 0.002 ^a^ 0.308 ± 0.003 ^a^ 0.320 ± 0.001 ^a^ 0.406 ± 0.004 ^b^	0.433 ± 0.009 ^b^ 0.443 ± 0.012 ^b^ 0.456 ± 0.014 ^b^ 0.463 ± 0.011 ^b^ 0.470 ± 0.018 ^b,c^	0.448 ± 0.014 ^b^ 0.534 ± 0.021 ^c^ 0.704 ± 0.028 ^d,e^ 0.760 ± 0.021 ^e^ 0.779 ± 0.024 ^e,f^	0.643 ± 0.003 ^d^ 0.745 ± 0.011 ^e^ 0.851 ± 0.019 ^f^ 0.859 ± 0.022 ^f^ 0.897 ± 0.025 ^f,g^	0.682 ± 0.009 ^d^ 0.868 ± 0.011 ^f^ 0.912 ± 0.021 ^g^ 0.983 ± 0.011 ^h^ 0.993 ± 0.017 ^h^	0.703 ± 0.011 ^d^ 0.959 ± 0.020 ^g,h^ 0.967 ± 0.018 ^g,h^ 0.986 ± 0.011 ^h^ 0.998 ± 0.006 ^h^
Adhesiveness, g·s	1 7 14 17 20	1477.9 ± 5.9 ^h^ 1368.3 ± 1.6 ^g^ 1291.5 ± 6.2 ^e^ 1227.0 ± 4.8 ^d^ 1212.8 ± 2.6 ^d^	1306.9 ± 5.7 ^f^ 1304.1 ± 3.6 ^f^ 1213.9 ± 2.9 ^d^ 1197.5 ± 2.3 ^c^ 1187.7 ± 4.8 ^c^	1299.3 ± 3.8 ^e,f^ 1293.3 ± 2.7 ^e^ 1200.6 ± 5.2 ^c,d^ 1186.7 ± 3.8 ^c^ 1175.3 ± 4.2 ^c^	1276.2 ± 4.6 ^e^ 1270.8 ± 3.1 ^e^ 1169.5 ± 2.8 ^b,c^ 1133.9 ± 2.5 ^a,b^ 1129.5 ± 3.3 ^a^	1232.1 ± 2.9 ^d^ 1223.1 ± 3.1 ^d^ 1134.7 ± 2.5 ^a,b^ 1129.5 ± 2.6 ^a^ 1114.0 ± 1.9 ^a^	1219.1 ± 6.2 ^d^ 1181.2 ± 5.4 ^c^ 1121.5 ± 4.7 ^a^ 1111.7 ± 6.1 ^a^ 1110.5 ± 2.9 ^a^
Gumminess, %	1 7 14 17 20	0.999 ± 0.002 ^j^ 0.965 ± 0.001 ^j^ 0.816 ± 0.006 ^h^ 0.801 ± 0.005 ^g,h^ 0.782 ± 0.004 ^g^	0.874 ± 0.004 ^i^ 0.845 ± 0.008 ^h^ 0.763 ± 0.010 ^g^ 0.771 ± 0.009 ^g^ 0.761 ± 0.006 ^g^	0.689 ± 0.005 ^f^ 0.669 ± 0.009 ^f^ 0.672 ± 0.011 ^f^ 0.642 ± 0.008 ^e,f^ 0.622 ± 0.013 ^e^	0.444 ± 0.011 ^d^ 0.438 ± 0.007 ^d^ 0.434 ± 0.009 ^c,d^ 0.424 ± 0.010 ^c,d^ 0.411 ± 0.007 ^c^	0.409 ± 0.006 ^c^ 0.405 ± 0.012 ^c^ 0.403 ± 0.009 ^c^ 0.377 ± 0.008 ^b,c^ 0.361 ± 0.011 ^b^	0.382 ± 0.012 ^b^ 0.372 ± 0.009 ^b,c^ 0.327 ± 0.005 ^a,b^ 0.319 ± 0.006 ^a,b^ 0.308 ± 0.011 ^a^

Values in the table represent the means of three replicated trials, ± standard deviation. Different letters (^a–k^) designate statistically different results (*p* ≤ 0.05).

**Table 8:** Syneresis evolution of yogurt with apple pomace powder during storage.

Parameter	Storage Period, Days	Samples					
		Y	0.2%YAP	0.4%YAP	0.6%YAP	0.8%YAP	1.0%YAP
Syneresis, %	1 7 14 17 20	26.65 ± 0.12 ^h^ 27.05 ± 0.32 ^i^ 27.39 ± 0.10 ^i^ 27.97 ± 0.04 ^j^ 28.13 ± 0.31 ^j^	25.72 ± 0.08 ^g^ 25.58 ± 0.11 ^g^ 24.89 ± 0.22 ^f^ 24.62 ± 0.09 ^e^ 24.46 ± 0.16 ^e^	24.94 ± 0.14 ^f^ 24.87 ± 0.12 ^f^ 24.33 ± 0.10 ^e^ 23.89 ± 0.09 ^d^ 23.63 ± 0.15 ^c,d^	24.55 ± 0.08 ^e^ 24.29 ± 0.05 ^e^ 23.96 ± 0.11 ^d^ 23.72 ± 0.07 ^c,d^ 23.16 ± 0.09 ^b,c^	24.48 ± 0.11 ^e^ 24.21 ± 0.09 ^d,e^ 23.61 ± 0.07 ^c,d^ 23.22 ± 0.05 ^c^ 22.53 ± 0.06 ^a^	24.38 ± 0.09 ^e^ 24.03 ± 0.07 ^d^ 23.39 ± 0.08 ^c^ 22.86 ± 0.11 ^b^ 22.18 ± 0.06 ^a^

Values in the table represent the means of three replicated trials, ± standard deviation. Different letters (^a–j^) designate statistically different results (*p* ≤ 0.05).

## Data Availability

No new data were created or analyzed in this study. Data sharing is not applicable to this article.
